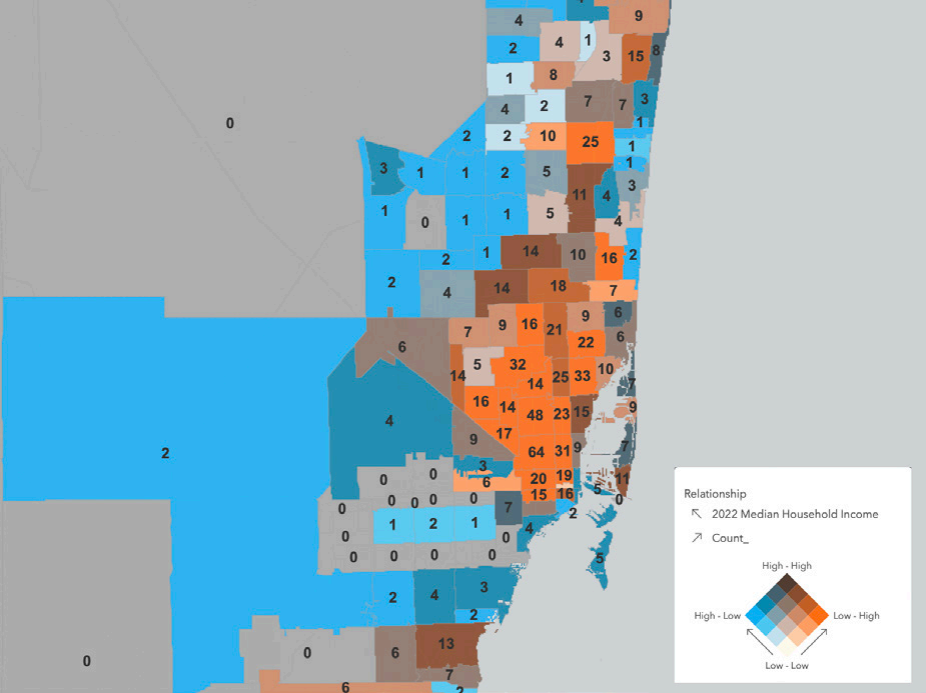# 37 The Epidemiology of Burn Injury in A Large Southern Geographic Area

**DOI:** 10.1093/jbcr/irad045.011

**Published:** 2023-05-15

**Authors:** Christopher Haggerty, Walter Ramsey, Christopher O'Neil, Shevonne Satahoo, Joyce Kaufman, Louis Pizano, Carl Schulman

**Affiliations:** University of Miami Miller School of Medicine, Miami, Florida; University of Miami, DeWitt Daughtry Family Department of Surgery - Division of Trauma, Acute Care Surgery, and Surgical Critical Care; Jackson Memorial Hospital - Ryder Trauma Center, Miami, Florida; University of Miami, Miami, Florida; University of Miami, Miami, Florida; University of Miami, Miami, Florida; University of Miami, Miami, Florida; University of Miami-Jackson Memorial Hospital, Miami, Florida; University of Miami Miller School of Medicine, Miami, Florida; University of Miami, DeWitt Daughtry Family Department of Surgery - Division of Trauma, Acute Care Surgery, and Surgical Critical Care; Jackson Memorial Hospital - Ryder Trauma Center, Miami, Florida; University of Miami, Miami, Florida; University of Miami, Miami, Florida; University of Miami, Miami, Florida; University of Miami, Miami, Florida; University of Miami-Jackson Memorial Hospital, Miami, Florida; University of Miami Miller School of Medicine, Miami, Florida; University of Miami, DeWitt Daughtry Family Department of Surgery - Division of Trauma, Acute Care Surgery, and Surgical Critical Care; Jackson Memorial Hospital - Ryder Trauma Center, Miami, Florida; University of Miami, Miami, Florida; University of Miami, Miami, Florida; University of Miami, Miami, Florida; University of Miami, Miami, Florida; University of Miami-Jackson Memorial Hospital, Miami, Florida; University of Miami Miller School of Medicine, Miami, Florida; University of Miami, DeWitt Daughtry Family Department of Surgery - Division of Trauma, Acute Care Surgery, and Surgical Critical Care; Jackson Memorial Hospital - Ryder Trauma Center, Miami, Florida; University of Miami, Miami, Florida; University of Miami, Miami, Florida; University of Miami, Miami, Florida; University of Miami, Miami, Florida; University of Miami-Jackson Memorial Hospital, Miami, Florida; University of Miami Miller School of Medicine, Miami, Florida; University of Miami, DeWitt Daughtry Family Department of Surgery - Division of Trauma, Acute Care Surgery, and Surgical Critical Care; Jackson Memorial Hospital - Ryder Trauma Center, Miami, Florida; University of Miami, Miami, Florida; University of Miami, Miami, Florida; University of Miami, Miami, Florida; University of Miami, Miami, Florida; University of Miami-Jackson Memorial Hospital, Miami, Florida; University of Miami Miller School of Medicine, Miami, Florida; University of Miami, DeWitt Daughtry Family Department of Surgery - Division of Trauma, Acute Care Surgery, and Surgical Critical Care; Jackson Memorial Hospital - Ryder Trauma Center, Miami, Florida; University of Miami, Miami, Florida; University of Miami, Miami, Florida; University of Miami, Miami, Florida; University of Miami, Miami, Florida; University of Miami-Jackson Memorial Hospital, Miami, Florida; University of Miami Miller School of Medicine, Miami, Florida; University of Miami, DeWitt Daughtry Family Department of Surgery - Division of Trauma, Acute Care Surgery, and Surgical Critical Care; Jackson Memorial Hospital - Ryder Trauma Center, Miami, Florida; University of Miami, Miami, Florida; University of Miami, Miami, Florida; University of Miami, Miami, Florida; University of Miami, Miami, Florida; University of Miami-Jackson Memorial Hospital, Miami, Florida

## Abstract

**Introduction:**

Given the resource-intense nature of treatment for burn injuries and the knowledge of at-risk populations, a public health approach to reducing individual and societal burden is indicated. To accomplish this, it is critical to understand the regional distribution of these injuries and the risk factors influencing one’s susceptibility to being burned. We hypothesize that there are common sociodemographic trends in burn injuries in a large southern geographic area.

**Methods:**

Data was retrospectively reviewed for all burn patients admitted between 08/2013 and 12/2021 at a major burn center in a large southern geographic area. Sociodemographic data, burn characteristics and outcomes were analyzed. Patients with isolated inhalation injuries were excluded. One-way ANOVA was used for continuous variables with a P-value < 0.05. Binary logistic regression was used to evaluate independent risk factors for mortality. GIS software mapped injury locations alongside census data to augment statistics with visuospatial analysis.

**Results:**

1753 patients were included with an annual incidence rate of 36 per 100,000 person-years. Most were male (68%), Caucasian (60%) and non-Hispanic (67%). The most common etiology was cooking-related (14%), with scald and flame burns collectively making up 80%. Flame burns were significantly larger when compared to scald, electrical and chemical mechanisms (p< 0.05). Those between 19 and 45 years old were the largest age group (40%) with significantly larger burns compared to those 0-18 and 46-64 (p< 0.05), but not those > 65. Non-Hispanic patients had significantly larger burns than their Hispanic counterparts (p< 0.05) but no difference in mortality (p=.306). Patients utilizing Medicare/Medicaid were over three times more likely to die compared to those with commercial insurance.

**Conclusions:**

In this cohort, male patients are injured at higher rates, however, they do not have more severe burns. The larger burns are distributed in a bimodal fashion between those 19-45 and those > 65, with hispanic ethnicity protective against larger burn injuries. This suggests separate mechanisms mediating larger burns in these two groups, and warrants further investigation. Insurance type, a surrogate for socioeconomic status (SES), is not associated with TBSA or LOS, however it does confer an increase in mortality in those with Medicare/Medicaid. GIS mapping verifies neighborhoods concentrated in more urban areas with lower median household incomes have higher injury rates. This highlights populations worth targeting for injury prevention, namely those between 19 and 45 of lower SES, particularly in the setting of cooking and food preparation.

**Applicability of Research to Practice:**

Understanding regional trends in burn injury can target prevention efforts and identify at risk populations where further resources might be best allocated to ease the burden of burn injury.